# Metabolomics as a promising technology for investigating external therapy of traditional Chinese medicine: A review

**DOI:** 10.1097/MD.0000000000040719

**Published:** 2024-11-29

**Authors:** Xinyue Yang, Min He, Qingqing Tang, Jiazhen Cao, Zhe Wei, Tie Li, Mengmeng Sun

**Affiliations:** a School of Medicine, Lishui University, Lishui, China; b Department of Acupuncture and Tuina, Changchun University of Chinese Medicine, Changchun, China; c Northeast Asia Research Institute of Traditional Chinese Medicine, Changchun University of Chinese Medicine, Changchun, China.

**Keywords:** acupuncture, metabolism pathway, metabolomics, moxibustion, TCM external therapy

## Abstract

To demonstrate the potential for connecting metabolomics with traditional Chinese medicine (TCM) external therapies such as acupuncture and moxibustion, we conducted a literature review on metabolomics as a measurement tool for determining the efficacy of various TCM external therapies. Human research and animal models published in the last 10 years were summarized. The investigation can be classified as follows: Using metabolomics to study metabolic profile changes produced by stimulation of a specific acupoint ST36 indicates the perturbation of metabolites produced by stimulation of acupoints by external TCM treatments can be characterized by metabolomics; and Using metabolomics to reveal the molecular mechanism of various TCM external therapy methods to treat specific diseases such as digestive system disease, cardiovascular disease, neurological disorder, bone disease, and muscle fatigue. We conclude that metabolomics has considerable potential for comprehending TCM external treatment interventions, particularly from a systems perspective. Linking TCM external therapy research with metabolomics can further bridge detailed biological mechanisms with the systematic effect of TCM external therapy, hence providing new paths for gaining a deeper knowledge of the importance of TCM in the treatment and maintenance of health.

## 1. Introduction

Traditional Chinese medicine (TCM) is a 3000-year-old system of prevention, diagnosis, treatment and health care practices.^[1]^ By taking an all-inclusive approach and treating each patient according to their unique set of symptoms, it has led to the development of a highly complex system for treating sickness and keeping one’s own health and well-being in tip-top shape.^[2]^ Over a long period of clinical practice, materialism and dialectics shaped the theoretical framework of TCM. TCM focuses on both disease treatment and disease prevention.^[3]^ TCM uses both external and internal therapies to treat and prevent disease. External therapy is the oldest kind of sickness treatment, with both broad and specific definitions, and it is still widely used. In its narrowest sense, external therapy refers to the application of specific techniques and drugs to the skin, orifices, meridians, acupoints and other parts of the human body in order to dredge meridians, run Qi and blood and balance Yin and Yang. In its broadest sense, external therapy includes all methods of disease treatment except oral herbal medicine, including acupuncture, electro-acupuncture, moxibustion, herb-partitioned moxibustion, acupoint catgut embedding, cupping, manual therapy etc.

Using TCM external therapy to prevent and treat disease, control illness, and preserve health has become a common practice around the world. But in contrast, modern scientific representation tools and systems for sickness knowledge are lacking in TCM external therapy,^[4]^ and the interpretation of its biological mechanisms remains a vital study subject. TCM external therapy has become an important topic in modern life science as a result of this development. TCM external therapy’s function and method have been bolstered by existing biological technology, particularly in the area of biological signaling pathways. TCM external treatment, on the other hand, has a systemic and holistic effect.^[5]^ It is challenging to correctly portray the effects of external treatment techniques on the complete body using a single signaling pathway-based research design and methodology. Research on TCM external therapy effects and mechanisms is thus aimed at developing research techniques and evaluation systems that are consistent with TCM’s inherent treatment characteristics and objectively evaluate the overall curative effect.

**Figure 1.**
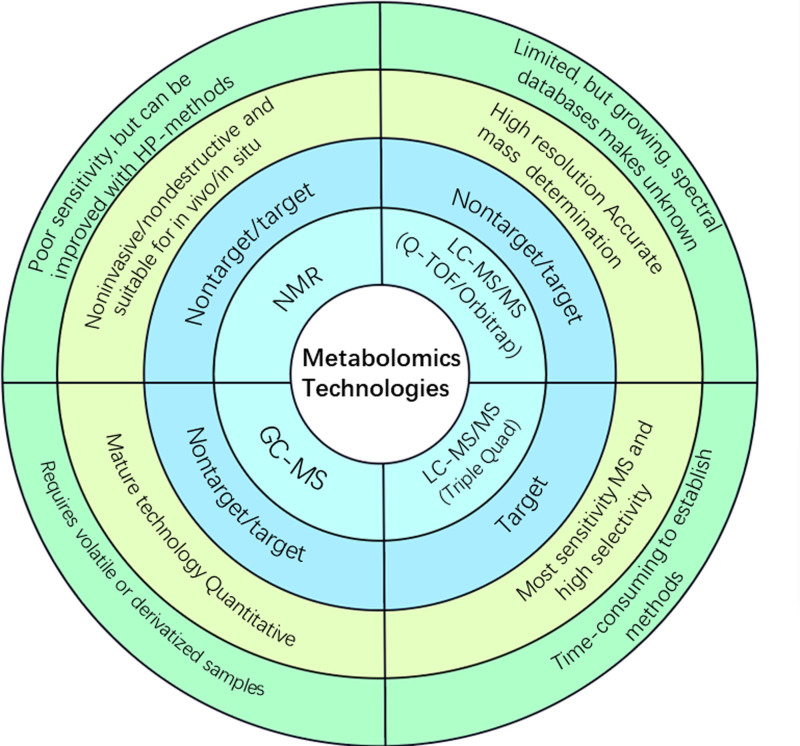
The characteristics of NMR, LC-MS, and GC-MS are briefly summarized. NMR and MS are 2 typical analytical platforms for metabolomics. Both NMR and MS techniques can automatically detect, identify, and measure metabolites with a high throughput. Metabolomics based on NMR and MS both have their own benefits and drawbacks. The approach is noninvasive and nondestructive, which is an advantage of NMR over MS. However, com-pared to MS-based techniques, the sensitivity of NMR is quite low and the metabolome coverage is quite limited. Gas chromatography mass spectrometry (GC-MS) requires volatile metabolites and gas-phase chemistry, whereas liquid chromatography mass spectrometry (LC-MS) approaches do not require volatile analyses and typically re-quire minimal sample preparation following extraction. Comparing LC-MS to GC/MS based on electron impact, LC-MS has less in-source fragmentation. NMR = nuclear magnetic resonance.

The biochemical phenotype of an organism’s general functioning condition based on the profile of small molecule metabolites in the body is known as “metabolomics.”^[6]^ An organism’s entire functional condition can be measured using metabolomics, which can be sensitive and objective in its reaction to a wide range of external stresses.^[7,8]^ Analytical approaches for metabolomics profiling include nuclear magnetic resonance (NMR) and mass spectrometric (MS).^[9]^ ^1^HNMR is the most often used NMR method. In addition to liquid samples, NMR spectroscopy can be applied to solid, gaseous, and tissue samples. One of its main advantages is the little sample preparation and preservation required (Fig. 1). The low instrument variability and high reproducibility of NMR are further notable features.^[10]^ The quantitative nature of this method allows direct comparisons of spectrum data, as it measures protons under a specific set of conditions.^[11]^ The main drawback of NMR is that its sensitivity is modest and its metabolome coverage is limited. (Fig. 1). Metabolomics is increasingly using LC-MS, a combination of liquid chromatography and mass spectrometry.^[12]^ Metabolites can be profiled using other techniques, such as gas chromatography linked to mass spectrometry (GC-MS) and capillary electrophoresis coupled to mass spectrometry. In general, MS has a larger sensitivity than NMR, allowing for the investigation of a broader range of metabolites (Fig. 1). Additionally, the various MS technologies offer a wide range of operational principles, expanding the number of possible metabolites that can be detected.

**Figure 2.**
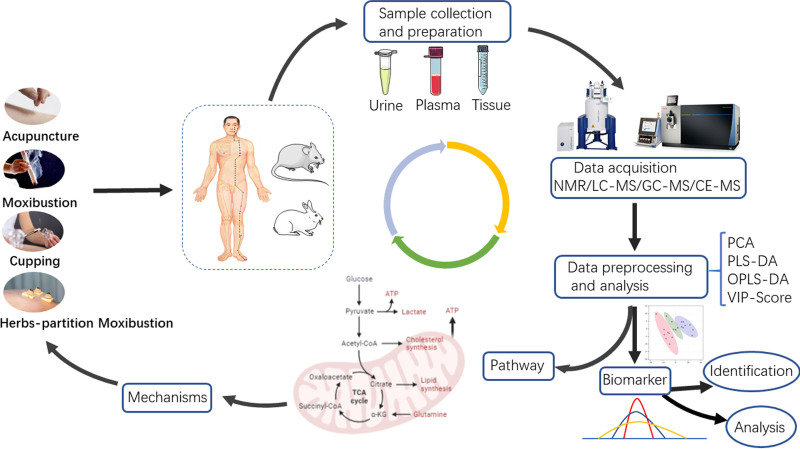
Application process of metabolomics in traditional Chinese Medicine.

In light of recent advances in metabolomics technology, many scientists believe that studying the metabolite profiles of living creatures is in keeping with the holistic, dynamic, and dialectical viewpoints of TCM.^[13]^ Now, biochemical causes and evidence based on variation in metabolite profiles are common in investigations on herbal efficacy and TCM diagnosis.^[14–18]^ The application of metabolomics in the research of the effects and mechanisms of TCM external treatment has also been increasing in recent years. Figure 2 depicts the metabolomics approach used in TCM research. To begin with, metabolomics investigations can be applied to nearly any external TCM therapy approach although the vast majority of these treatments are for acupuncture and moxibustion. Human and animal samples were used in the research. Samples of urine, blood, and other human and animal tissues are included in this collection. NMR and LC-MS are the most commonly used metabolomics research equipment for TCM external therapy. Multivariate statistical analysis is used to identify differential metabolites once the detection data is processed. Next, the Kyoto Encyclopedia of Genes and Genomes metabolic pathway was used to study these metabolites in order to explain the molecular mechanism of TCM external therapy’s therapeutic impact. In order to provide a complete summary of the studies that combine metabolomics and TCM external therapy, we have outlined this review. In addition, this evaluation will serve as a springboard for future research into TCM external therapy and inspire scholars interested in this field.

## 2. Metabolomics reflects changes in metabolites profile after stimulation of acupoint ST36

ST36 is a critical acupoint in TCM external therapy, both in disease treatment and daily health care.^[19–21]^ TCM defines a healthy body as one in which the yin and yang energies are in harmony and the Qi and blood flow freely.^[22]^ Stimulating ST36 in a healthy state can help maintain balance of energy in the body. Metabolomics has showed considerable potential as a technique to assess biological impacts of acupoints by capturing global alterations and overall physiological condition in biochemical networks and pathways.^[23]^ For example, stimulating on ST36 by acupuncture and moxibustion can affect several core metabolites and metabolic pathways connected to energy metabolism, which corresponds to the biological effects of ST36 in TCM that regulate Qi and blood flow and cause energy shifts.

According to the research shown in Table 1, activating ST36 under physiologically normal settings can cause biological impacts of metabolite alterations.^[24–31]^ In these research, whatever using rats or human’s samples, such as blood, saliva, stomach and liver tissue, or different metabolomics techniques, for instance, ultra performance liquid chromatography (UPLC)/ESI-Q-TOF/MS or ^1^H NMR, to study biological mechanism of activating ST36. The biological mechanism is all related to the metabolism pathways of amino acids and fatty acids.^[24,27–29]^ Amino acid metabolism is the process whereby protein meals are converted into tissue proteins and then broken down to provide energy in the body. Cellular uptake of fatty acids regulates the overall regulation of their metabolism, where they may act as precursors for the synthesis of other chemicals or as fuels for energy production. The bodies of ketone can then be transported to different tissues and utilized for energy generation. To put it another way, the processes of metabolizing amino acids and fatty acids are linked to those of metabolizing energy.

**Table 1:** Selected metabolomics studies in stimulation of acupoint ST36

Acupoint	Model system	Tissue	Platform	Therapy	Metabolic pathway	Findings	Ref
ST36	Human	Urine	UPLC/ESI-HDMS	Acupuncture	Amino acid metabolism	The physiological effect of needling ST36 can be determined by the fluctuation of 53 distinct metabolites in 5 classical amino acid metabolism pathways.	^[24]^
	Human	Saliva	UPLC/ESI-HDMS	Acupuncture	Amino acid metabolism; Steroid hormone metabolism	The physiological effects of needling ST36 can be explained by 26 different metabolites linked to the metabolism of amino acids and steroid hormones.	^[25]^
	Human	Serum	UPLC/ESI-Q-TOF/MS	Acupuncture	Glycerophospholipid metabolism; Ether lipid metabolism; Fatty acid metabolism; Glycerolipid metabolism; Porphyrin metabolism; Sphingolipid metabolism; Primary bile acid biosynthesis; Fatty acid elongation in mitochondria; Fatty acid biosynthesis; Tryptophan metabolism	The physiological effects of needling ST36 can be explained can be explained by 34 different metabolites linked to the metabolism of 10 metabolism pathways.	^[26]^
	Human	Serum	^1^H NMR	Moxibustion	Amino acids metabolism; Fatty acid metabolism	Moxibustion has been shown to increase the levels of branched-chain amino acids, polyunsaturated fatty acids, and other metabolites, so enhancing the body’s amino acid and fatty acid metabolisms.	^[27]^
	Rats	Serum; Urine; Cortex; Stomach	^1^H NMR	Electro-acupuncture	Amino acid metabolism; Fatty acid metabolism; Choline metabolism;	Electro-acupuncture therapy significantly altered 31 metabolites, including amino acids, organic acids, choline esters, and glucose.	^[28]^
	Rats	Cortex; Liver; Stomach; Plasma	^1^H NMR	Electro-acupuncture; Moxibustion	Amino acid metabolism	Electro-acupuncture and moxibustion both altered the metabolome of the cortex and raised the levels of phenylalanine, tyrosine, and branched-chain amino acids in the liver. Electro-acupuncture has been shown to boost ATP levels in the brain, creatine and dimethylglycine levels in the stomach, and GABA levels in the liver. Moxibustion raised the plasma levels of enkephalin, as well as the concentrations of betaine and fumarate in the stomach.	^[29]^
	Rats	Heart and stomach tissues	LC–MS	Electro-acupuncture	Amino acid metabolism	Ten metabolites were upregulated and 7 were downregulated as a result of electroacupuncture at ST36. The stomach tissues of rats in the ST36 group revealed that electro-acupuncture at ST36 led to the overexpression and downregulation of 10 and eighteen metabolites, respectively.	^[30]^
	Rats	Brain	LC–MS	Electro-acupuncture	Amino acid metabolism and neurotransmitters	Electroacupuncture at ST6 resulted in the elevation of adrenaline and arginine in the hippocampus, but stimulations in the hypothalamus had minimal effects on identified neurotransmitters.	^[31]^

LC-MS = liquid chromatography-mass spectrometry, NMR = nuclear magnetic resonance.

Other metabolic pathways which were affected by activating ST36, such as steroid hormone metabolism, glycerophospholipid metabolism and ether lipid metabolism,^[25,26]^ are also revealed to the effect of TCM external therapy. The adrenal gland and gonads are the primary sources of steroid hormone production. Energy metabolism, stress response, salt balance and sexual function are all controlled by these hormones. Key chemicals in cell structure and control, such as phospholipids, are critical to understanding how cells function. This metabolic process is a crucial part of cell growth and development. The fatty acid metabolism is the subject of the following metabolic pathways. Thus, they’re all linked to the processes that produce and use energy.

Moreover, researchers stimulated ST36 and PC6 acupoints, respectively, in animal model and employed LC-MS-based metabolomics to examine the metabolic consequences generated by activating distinct acupoints.^[30,31]^ When ST36 and PC6 were activated, the metabolic pathways regulated by activation of ST36 and PC6 were considerably different. Therefore, using metabolomics to examine the TCM concept of acupoint specificity, it can be shown that stimulating distinct acupoints can have diverse physiological functions and therapeutic benefits.

## 3. Metabolomics for assessing the efficacy of TCM external therapy for disease

### 3.1. Digestive system disease

In the treatment of digestive disorders, the more widely used external TCM treatments are acupuncture, electroacupuncture and moxibustion. Functional gastrointestinal disorders are more frequently treated with electroacupuncture and acupuncture. Acupuncture prescriptions composed of several acupuncture sites have long been the focus of clinical therapy. For example, activating the ST21-ST36 combination is often used to treat gastrointestinal issues. It is possible to use ST21 and ST36 together to treat chronic atrophic gastritis (CAG) in a synergistic manner that has a greater impact on the metabolic network than a simple additive effect. An inflammation of the digestive system known as CAG causes the body’s immune system to wrongly destroy the parietal cells in the stomach. Several researchers investigated whether acupuncture and moxibustion affect CAG rats in a positive way. ST21 and ST36 are the primary acupoints used in the treatment of acupuncture and moxibustion,^[32–34]^ as showed in Table 2. Blood, urine, stomach tissues and other organ tissues are the study samples for metabolomics analysis. Electro-acupuncture and moxibustion restored several CAG-induced metabolic alterations including membrane metabolism, energy metabolism, and neurotransmitter function in all sample types.^[35]^ Electro-acupuncture and moxibustion both have an important function in the treatment of CAG, but the regulation effect of electro-acupuncture is more focused on the stomach and brain nerve systems.^[33]^ The significance of moxibustion in regulating hepatic energy is essential.^[37]^ To a certain extent, alterations of metabolites and metabolic pathways can be used to explain the therapeutic benefits of stimulating various acupoints in the body through the lens of metabolomics. The mechanism by which acupuncture and moxibustion vary in treating sickness can be studied in this way. Furthermore, it shows that metabolomics may be used to study the effects of different acupuncture systems in a complete and systematic manner.

**Table 2:** Selected metabolomics studies in digestive system disease

Disease	Model system	Tissue	Platform	Therapy	Acupoint	Metabolic pathway	Findings	Ref
Chronic atrophic gastritis (CAG)	Rats	Urine; Serum; Stomach; Cortex; Medulla	^1^H NMR	Electro-acupuncture	ST2 ST21 ST36	Energy metabolism; Membrane metabolism	Electro-acupuncture treatment normalizes the metabolomics abnormalities in CAG by reversing membrane catabolism, restoring neurotransmitter function in the brain.	^[35]^
	Rats	Serum; Stomach; Cerebral cortex; Medulla oblongata	^1^H NMR	Moxibustion	ST21 ST36	Amino acid metabolism; Energy metabolism; Fatty acid metabolism; Glucose metabolism	Moxibustion’s effect on CAG is manifested by changes in metabolites involved in amino acid, energy, fatty acid, and glucose metabolism.	^[32]^
	Rats	Serum; Stomach; Cerebral cortex; Medulla	^1^H NMR	Electro-acupuncture; Moxibustion	ST21 ST36	Membrane metabolism; Energy metabolism	Electro-acupuncture and moxibustion both restore metabolic alterations in CAG that are implicated in membrane metabolism, energy metabolism, and neurotransmitter function. Moxibustion was critical in CAG treatment because it regulated energy metabolism, whereas electro-acupuncture treatment acted mostly on the neurological system.	^[33]^
	Rats	Liver; Kidney	^1^H NMR	Acupuncture; Moxibustion	ST21 ST36	Energy metabolism; Neurotransmitter metabolism; Antioxidation metabolism	Acupuncture and moxibustion both have a beneficial effect on CAG. Moxibustion was more effective than electroacupuncture in regulating the energy metabolism of the liver.	^[34]^
	Rats	Gastric tissue	^1^H NMR	Acupuncture; Moxibustion	ST36 RN12	Amino acid metabolism	Moxibustion and acupuncture have distinct impacts on the metabolism of stomach tissue in CAG rats. Moxibustion modulation promotes stomach mucosal healing and immunity in CAG rats exposed to GSH, NAA, PC and Ura.	^[36]^
Gastric ulcer	Rats	Serum; Urine	^1^H NMR	Electro-acupuncture	ST2 ST21 ST36 GB14 GB24 GB34	Amino acid metabolism; Fatty acid metabolism; Glucose metabolism	Electro-acupuncture is effective to the repair of the stomach mucosa, as indicated by changes in metabolites involved in amino acid, fatty acid, and glucose metabolic pathways.	^[37]^
	Rats	Gastric mucosa tissue	^1^H NMR	Electro-acupuncture	ST21 ST36 GB24 GB34	Amino acid metabolism; Energy metabolism; Fatty acid metabolism	Electro-acupuncture aids in the repair of the stomach mucosa, as indicated by changes in metabolites involved in amino acid, energy, and fatty acid metabolic pathways.	^[38]^
	Rats	Stomach; Liver; Kidney	^1^H NMR	Electro-acupuncture	ST21 ST36	Energy metabolism; Cells metabolism	Electro-acupuncture induced changes in a wide variety of metabolic processes in the gastric ulcer, including neurotransmitter function, energy metabolism, cell metabolism, anti-oxidation, tissue healing, and other metabolic pathways.	^[39]^
	Rats	Serum; Urine	^1^H NMR	Electro-acupuncture	ST21 ST36	Amino acid metabolism; Fatty acid metabolism	Electro-acupuncture can aid in the repair of gastric mucosal injury in rats with gastric ulcers by modulating amino acid and fat metabolism.	^[40]^
	Rats	Urine; Serum	^1^H NMR	Electro-acupuncture	ST2 ST21 ST36 GB14 GB24 GB34	Amino acid metabolism	The effects of electro-acupuncture were mirrored in the alterations in metabolites related to amino acid metabolism.	^[41]^
	Rats	Stomach; Cerebral cortex; Medulla	^1^H NMR	Electro-acupuncture	ST36 ST21	Energy metabolism; Neurotransmitters; Membrane metabolism; Antioxidation	There were several possible metabolites discovered to engage in the pathophysiology and regulation of electro-acupuncture, with 15 in the medulla and 10 in the cerebral cortex.	^[42]^
	Rats	Serum; Stomach issue	^1^H NMR	Moxibustion	ST21 ST36	Energy metabolism; Glucose metabolism; Purine metabolism; Amino acid metabolism; Phospholipid metabolism	Moxibustion has a therapeutic effect on stomach ulcers and modulates metabolic abnormalities such as energy, glucose, purine, amino acid, and phospholipid metabolism.	^[43]^
Functional dyspepsia	Human	Plasma	^1^H NMR	Acupuncture	Six acupoints, all belonging to the Stomach Meridian of Foot-Yangming	Amino acid metabolism; Fatty acid metabolism; Glucose metabolism	Acupuncture treatment of FD patients resulted in considerable changes in leucine/isoleucine, lactate, and glucose levels, but only a minor improvement in lipid levels.	^[44]^
	Human	Plasma	^1^H NMR	Electro-acupuncture	RN12	Lactic acid metabolism; Amino acid metabolism; Glucose metabolism	After electroacupuncture the changes of metabolites were more significant in the 4 courses of treatment related to 10 differential metabolites.	^[45]^
	Rats	Serum	^1^H NMR	Electro-acupuncture	BL21 CV12	Fatty acid metabolism	Electro-acupuncture can be used to counteract or alleviate the effects of FD on VLDL/LDL cholesterol levels and blood NAc levels.	^[46]^
Functional constipation	Human	Serum	^1^H NMR	Electro-acupuncture	LI11 ST37	Fatty acid metabolism; Amino acid metabolism	Metabolites associated with lipid metabolism and amino acid metabolism pathways were altered following low-intensity electro-acupuncture.	^[47]^
Ulcerative colitis	Mice	Serum	HPLC-MS	Moxibustion	ST36 RN4	Fatty acid metabolism; Amino acid metabolism; Energy metabolism	Moxibustion has been shown to be effective in treating ulcerative colitis by modulating fatty acid, amino acid, and energy metabolism.	^[48]^
	Rats	Urine	^1^H NMR	Electro-acupuncture	ST36 RN4	No specific metabolic pathway	Electro-acupuncture has a protective impact on the mucosa of the intestine. and can facilitate the cell’s energy utilization. The main metabolites of acupuncture regulation include 3-D-hydroxybutyrate, hippurate, and acetate.	^[49]^
Irritable bowel syndrome	Rats	Fecal; Serum	^1^H NMR	Herb-partitioned moxibustion treatment	BL18 BL20 ST36 LR13 LA14	Amino acid metabolism; Bile acids metabolism	Herb-partitioned moxibustion was found to be effective for normalizing a number of IBS-induced metabolic abnormalities involving amino acid metabolism and bile acids.	^[50]^

NMR = nuclear magnetic resonance.

An open sore on the stomach’s lining is known as a gastric ulcer (GU). A burning or gnawing ache in the core of the belly is the most prevalent symptom of a stomach ulcer. Relieving pain is an important benefit of acupuncture and moxibustion, as well as aiding in the repair of gastrointestinal tissue. Some researchers employ the standard acupoint selection method, which combines ST21 and ST36,^[39,40,42,43]^ while others add GB24 and GB34,^[37,38,41]^ which are also beneficial for digestive system illnesses, showing in Table 2. The ^1^H NMR analysis of blood, urine, and tissue samples revealed that acupuncture and moxibustion impacted several metabolic pathways, including the metabolism of amino acids, energy, and fatty acids etc. Although the metabolic pathways detected are similar in those researches, this may be due to the non-targeted detection of ^1^H NMR, which is reflected in the same category of metabolic pathways; on the other hand, these similar changes may be related to the stimulation of ST36. In addition, Wei et al^43]^ administered moxibustion to GU rats, and ^1^H NMR based metabolomics revealed that moxibustion affected not only the aforementioned metabolic pathways, but also purine metabolism and phospholipid metabolism. This is due to the abnormal function of the tricarboxylic acid cycle and the creatine phosphate creatine shuttle system in GU, as well as the disorder of energy metabolism, which will promote the secondary disorder of glucose metabolism and the imposition. In terms of energy regulation, moxibustion may be superior to acupuncture, which is consistent with the distinction between acupuncture and moxibustion’s therapeutic efficacy as represented in CAG.

For functional dyspepsia, most of the metabolic pathways that are changed by acupuncture have close relationship to the metabolism of amino acids, fatty acids, and glucose.^[44–46]^ In addition, ^1^H NMR was used to find metabolic changes in human functional constipation patients who received electro-acupuncture treatment.^[47]^ It was found that electro-acupuncture also affected fatty acids and amino acids metabolism. After treating ulcerative colitis with moxibustion,^[48]^ which combines ST36 and RN4, animal blood samples were detected by high performance liquid chromatography-MS based metabolomics. It was found that the affected metabolic pathway was related to fatty acid, amino acid and energy metabolism. Moreover, it was found that electro-acupuncture has a positive regulation effect on key metabolites like 3-d-hydroxybutyrate, Hippurate, and acetate in ulcerative colitis model rats.^[49]^ Irritable bowel syndrome (IBS) is a functional disease of the intestines. Lin et al^[50]^ used herb-partitioned moxibustion to treat IBS model rats. Herb-partitioned moxibustion cures diseases by putting a piece of herb formula on the acupoints and then lighting a moxibustion tube near the herb formula in order to obtain synergistic treatment effect between herb and moxibustion. It was found that the regulation of amino acid metabolism and bile acid metabolism may be the molecular causes of how the herb-partitioned moxibustion treatment works on IBS.

### 3.2. Cardiovascular diseases

Hypertension can be effectively treated with acupuncture and moxibustion. More than a few clinical trials have shown that the use of active acupoints in hypertension is an effective way to treat hypertension. TCM’s active acupoints are those that are chosen based on the theory of meridians and acupoints and have a positive impact on sickness. When it comes to hypertension, ST9 is one of the most important active acupoint for treatment. Urine samples were studied using ^1^H NMR based metabolomics in Wang’s investigation on the effects of acupuncture ST9 on a hypertension rat model.^[51]^ Acupuncture at ST9 raised the levels of α-ketoglutaric acid, N-acetylglutamic acid, and betaine in the urine, and resulted in significant decreases in blood pressure, heart rate, and mean arterial pressure.

The biological proof of the difference between active and inactive acupoints in the treatment of hypertension was also being studied. Yang et al^[52]^ investigated the clinical efficacy of acupuncture at active (such as LR3, KI3, PC6, and PC9) and non-active acupoints in patients with hypertension, and analyzed the blood samples of those patients using LC-MS based metabolomics. According to the results of the clinical trials, acupuncture on active acupoints dramatically reduced 24-hour systolic blood pressure but had no effect on diastolic blood pressure. Metabolomics data from the active and non-active acupoints treatment groups show substantial differences, revealing 2 separate post-treatment metabolic profiles. In another study,^[53]^ it was found that acupuncture reduced 24-hour systolic and diastolic blood pressure and improved the circadian rhythm of blood pressure. These positive changes were associated with changes in patient blood metabolites. These investigations have successfully proved the therapeutic effect of stimulating acupoints on a disease in the case of hypertension. Although these studies only reflect changes in the metabolites of hypertension before and after treatment, there is no metabolic pathway analysis, which shows that the regulated metabolic pathways of stimulating acupoints have to be further researched in hypertension.

Additionally, hyperlipidemia and atherosclerosis are very prevalent cardiac system illnesses. An elevated amount of LDL-C (the bad cholesterol) and a decrease in HDL-C (the good cholesterol) are all signs of hyperlipidemia, a disorder in which the body’s lipid metabolism is impaired (HDL-C). Patients with hyperlipidemia are treated by moxibustion, and their blood samples were studied using LC-MS based metabolomics analysis.^[54]^ Moxibustion was reported to effectively alleviate hyperlipidemia, possibly through altering the levels of TC and TG, and regulating glycerophospholipid metabolism and sphingolipid metabolism pathways. Another research showed that electroacupuncture reduced carotid artery lipid accumulation and successfully regulated differential metabolites linked to lipid metabolism in atherosclerosis model rabbits.^[55]^

Electro-acupuncture has been shown to have a positive effect on chronic myocardial ischemia. There are significant alterations in myocardial energy metabolism during ischemia because of the decreased oxygen supply. In some cases, these metabolic alterations are advantageous because they aid the heart in its recovery from ischemia. ST36, CV4, and PC6 are all acupoints that can be stimulated using electroacupuncture for treatment of chronic myocardial ischemia^[56,57]^ (Table 3). According to a ^1^H NMR based metabolomics study conducted on rats, electro-acupuncture not only helped mend the myocardium, but it also altered the differential metabolites associated to lipid and energy metabolism in the rats. When the myocardial ischemia reperfusion injury rats’ model was investigated, Chen et al found that pyruvate, amino acid and energy metabolism were regulated by electro-acupuncture in the model rats.^[58]^

**Table 3:** Selected metabolomics studies in cardiovascular diseases

Disease	Model system	Tissue	Platform	Therapy	Acupoint	Metabolic pathway	Findings	Ref
Hypertension	Human	Plasma	MRM-MS	Acupuncture	LR3 ST9 KI3 PC6 ST36 LI11	No specific metabolic pathway	Acupuncture has a beneficial effect on hypertension, and the most significant differential metabolites after acupuncture treatment were oleic acid and myoinositol.	^[53]^
	Rats	Urine	^1^H NMR	Acupuncture	ST9	No specific metabolic pathway	Acupuncture has been shown to lower blood pressure by correcting the metabolic problem associated with hypertension as measured by 46 metabolites.	^[51]^
	Human	Plasma	LC-MS	Acupuncture	LR3 ST9 KI3 PC6 GB20 SJ5 SP9 SP10	No specific metabolic pathway	Acupuncture on active acupoints has been shown to reduce blood pressure. The most important metabolites of active acupoint treatment are sucrose, cellobiose, and hypoxanthine.	^[52]^
Hyperlipidemia	Human	Plasma	LC-MS	Moxibustion	ST40	Glycerophospholipid metabolism; Sphingolipid metabolism	Moxibustion effectively improved hyperlipidemia, possibly by influencing TC and TG levels and controlling the glycerophospholipid metabolism and sphingolipid metabolism pathways.	^[54]^
Atherosclerosis	Rabbit	Plasma	HPLC-TOF-MS	Electro-acupuncture	PC6 ST36 BL26	Lipid metabolism	Electro-acupuncture decreased lipid accumulation on the carotid artery wall and regulated lipid metabolism.	^[55]^
Chronic Myocardial Ischemia	Rats	Plasma	^1^H NMR	Electro-acupuncture	ST36 CV4 PC6	Lipid metabolism; Energy metabolism.	Electro-acupuncture has been shown to improve ischemic myocardial damage, which may be due to its effect on serum sugar, lipid, and energy metabolism.	^[56]^
	Rats	Plasma	^1^H NMR	Electro-acupuncture	ST36 CV4 PC6	Energy metabolism; Lipid metabolism; Glucose metabolism; Amino acid metabolism.	Electro-acupuncture has been shown to improve ischemic myocardial damage, which may be due to its effect on serum sugar, lipid, and energy metabolism.	^[57]^
Myocardial ischemia reperfusion injury	Rats	Plasma	^1^H NMR	Electro-acupuncture	PC6	Pyruvate metabolism; Amino acid metabolism; Ketone body metabolism; Energy metabolism	Electro-acupuncture has a protective effect by regulating gluconeogenesis, pyruvate metabolism, amino metabolism, ketone body metabolism and energy metabolism.	^[58]^

LC-MS = liquid chromatography-mass spectrometry, NMR = nuclear magnetic resonance, TC = total cholesterol, TG = triglyceride.

### 3.3. Neurological disorders

Alzheimer’s disease (AD) is a slowly progressive neurological ailment marked by memory loss, cognitive impairment, and personality changes. Metabolic disorders, such as lipid, glucose and amino acid metabolism, may pose a significant threat to the progression of AD. Recent research indicated that acupuncture treatment significantly improved the learning and memory capacity of AD model mice. The metabolic alterations in liver, kidney, and blood samples from AD animal models treated with electro-acupuncture have been studied using ^1^H NMR-based metabolomics^[59,60]^ (Table 4). The impacted metabolic changes were discovered to be connected to the aforementioned pathways. Some other research investigated the effect of moxibustion on mice with AD, and evaluated mice’s urine samples using UPLC-MS based metabolomics^[61,62,64]^ (Table 4). The results showed that moxibustion can control substance and energy metabolism by enhancing the production and breakdown of carbs and amino acids. Acupuncture, being a holistic and systemic treatment, can not only regulate metabolites but also affect the quantity of intestinal microbes. Analysis of feces samples from the APP/PS1 mice showed a substantial impact on the abundance changes of gut microbiota as well as metabolites in the presence of an acupuncture therapy.^[63]^ It should be emphasized that different acupuncture prescriptions are used in these AD-related investigations. This is due to the fact that, under the supervision of TCM theory, clinicians identify the symptom kinds of the same disease and select acupoints and prescriptions for distinct TCM syndrome types, which also represents the diverse treatment of the same disease in TCM theory. Metabolomics may further provide biological foundation for this special TCM external therapy theory.

**Table 4:** Selected metabolomics studies in neurological disorders

Disease	Model system	Tissue	Platform	Therapy	Acupoint	Metabolic pathway	Findings	Ref
Alzheimer’s disease	Mice	Liver	^1^H NMR	Electro-acupuncture	GV20 KI 1	Energy metabolism; Lipid metabolism; Glucose metabolism; Amino acid metabolism.	Electro-acupuncture can regulate many metabolites associated with energy metabolism, lipid metabolism, glucose metabolism, and amino acid metabolism in the livers.	^[59]^
	Mice	Plasma; Liver; Kidney	^1^H NMR	Electro-acupuncture	GV 20 KI 1	Lipid metabolism; Glucose metabolism; Fatty acids metabolism.	Electro-acupuncture has been shown to alter the metabolite spectrum of plasma, liver, and kidney in SAMP8 mice associated with lipid, glucose, and fatty acid metabolism pathways.	^[60]^
	Mice	Urine	UPLC-MS	Moxibustion	CV4	Amino acid metabolism; Sucrose metabolism; Nitrogen metabolism	Moxibustion has been shown in AD model mice to alter the levels of key biomarkers in metabolic pathways such as amino acid, starch, sucrose, mutual transformation of pentose and glucuronic acid vinegar, nitrogen metabolism, and ammonia production.	^[61]^
	Mice	Urine	UPLC-MS	Moxibustion	CV4	Amino acid metabolism; Starch metabolism; Sucrose metabolism; Glucuronate metabolism	Moxibustion has been shown to improve carbohydrate and amino acid production and breakdown in APP/PS1 transgenic AD model mice.	^[62]^
	Mice	Fecal	LC-QTOF-MS	Acupuncture	DU20 LI4 BL13 BL20 BL23 ST36 SP6	Pyrimidine metabolism; Linoleic acid metabolism; Amino acid metabolism; Phenylalanine metabolism	Acupuncture treatment had been shown to impact the microbiota and metabolites of mice and to be associated with 6 biological pathways.	^[63]^
	Mice	Plasma	^1^H NMR	Electro-acupuncture	GV20 KI 1	No specific metabolic pathway	Electro-acupuncture may have the ability to mitigate neurodegeneration and protect the brain by altering the molecular weight of certain metabolites, including lactate, trimethylamine N-oxide, dimethylamine, and choline.	^[64]^
Chronic stress	Rats	Plasma	^1^H NMR	Electro-acupuncture	GV20 SP6	Glucose metabolism; Lipid metabolism	Electro-acupuncture has a considerable effect on the metabolites associated with glucose and lipid metabolism in chronic emotional stress and anxiety model mice.	^[65]^
	Rats	Plasma	^1^H NMR	Electro‑acupuncture	ST36	Glucose metabolism	Electro-acupuncture’s effect on chronic stress is most likely due to its ability to regulate glucose metabolism, which can serve as a model for clinical acupuncture therapy of chronic stress depression.	^[66]^
Depression	Mice	Liver	UPLC/Q-TOF-MS	Acupuncture	KI10 LR8 LU8 LR4	Lipid metabolism	Acupuncture treatment significantly decreased depressive-like behaviors, altered immune responses, and improved hepatic lipid metabolism by attenuating leptin resistance.	^[67]^
	Mice	Brain tissue	LC-MS/MS	Acupoint Catgut Embedding	GV20 GV14	Vitamin B6 metabolism; Retinol metabolism	Acupoint catgut embedding therapy alleviated depression-related symptoms in depressed rats by reversing metabolic abnormalities in the brain.	^[68]^
Acute incomplete spinal cord injuries	Rats	Cerebral cortex	^1^H NMR	Electro-acupuncture	BL54 ST28	Amino acid metabolism; Lipid metabolism	Electro-acupuncture is beneficial for spinal cord injury rehabilitation, as demonstrated by changes in metabolites linked with amino acid and lipid metabolism in the cerebral cortex.	^[69]^
Acute Migraine	Rats	Plasma	^1^H NMR	Electro-acupuncture	SJ5 GB34	Lipids metabolism; Glutamate metabolism	Electro-acupuncture at acupoints may alleviate acute migraine by restoring the plasma metabolic profile and plasma glutamate.	^[70]^
Ischemic stroke	Rats	Plasma	LC/MS	Acupuncture	LI11 GB34 ST36	Isoflavones phyto-estrogens metabolites	The efficiency of acupuncture combined with a herbal formula was superior to that of a single treatment for ischemic stroke; the combination therapy not only corrected gut microbiota imbalances and increased the number of beneficial bacteria, but also alleviated metabolic problems.	^[71]^

AD = Alzheimer’s disease, LC-MS = liquid chromatography-mass spectrometry, NMR = nuclear magnetic resonance.

Similar to AD, chronic stress is a disorder of the neurological system. It has to do with the glucose metabolism. Hypermetabolism characterized by increased glycolysis, gluconeogenesis, altered glucose absorption, and decreased glycogenesis has been linked to chronic stress. Chronic stress is associated with the development of chronic illness and metabolic abnormalities. As a result of long-term allostasis, chronic long-term stress generated abnormalities in glucose metabolism and the development of insulin resistance and glucose intolerance.^[72]^ In recent research, Wu et al^[65]^ and Ma et al^[66]^ demonstrated that electro-acupuncture had a positive effect in rats with chronic stress. With the detection of blood samples by ^1^H NMR based metabolomics, it was discovered that electro-acupuncture effectively modulated the glucose metabolism of model animals.

Depression can also negatively alter lipid metabolism. Consequently, a number of studies have demonstrated a link between depression and higher triglyceride (TG) and low high-density lipoprotein cholesterol (HDL-C) levels.^[73]^ Jung et al^[67]^ tested the treatment effect of acupuncture on mice modeled after depression. They found that acupuncture helps alleviate anxious behavior, which may be related to lipid metabolism changes. Duan et al^[68]^ applies catgut acupoint embedding to treat the same model animals. Acupoint catgut embedding refers to the process of embedding absorbable sutures into the skin tissue of acupoints intimately associated with various physiological processes or disorders. Acupoint catgut embedding reduced symptoms of depression in model rats by rectifying metabolic anomalies in the brain related to the metabolism of vitamin B6 and retinol. In general, catgut embedding therapy is a popular way of treatment in recent years. However, there are currently very few scientific studies that provide evidence for this treatment strategy. Metabolomics might be considered as a tool to support this treatment in the future.

In addition, Quan et al^[69]^ examined the effects of electro-acupuncture on acute incomplete spinal cord injuries and discovered that it is useful for spinal cord injury rehabilitation, as evidenced by changes in metabolites associated with amino acid and lipid metabolism in the cerebral cortex. Moreover, Gao et al^[70]^ chose mice as disease models for the research on acute migraine. They found that electro-acupuncture may treat acute migraine by correcting the metabolic profile and glutamate levels of plasma. Furthermore, the efficiency of acupuncture combined with a herbal formula was superior to that of a single treatment for ischemic stroke^[71]^; the combination therapy not only corrected gut microbiota imbalances and increased the number of beneficial bacteria, but also alleviated metabolic problems.

### 3.4. Bone disease and muscle fatigue

As the most common inflammatory arthritis, rheumatoid arthritis (RA) is maintained by immune system responses.^[74]^ Immune responses, both innate and adaptive, are tightly controlled by amino acid metabolism in mammals. A disruption in the pyrimidine metabolic pathway is one of the hallmarks of RA.^[75]^ Meanwhile, arthritic joints and rheumatoid arthritis appear to be metabolizing glucose more quickly than healthy joints.^[76]^ Patients with RA can use blood samples to monitor their disease progression.^[77]^ Patients with RA treated with acupuncture participated in a metabolomic study by Guo et al using GC-MS to analyze blood samples.^[78]^ Tenderness, swelling, and morning stiffness were all reduced after acupuncture treatment, and the metabolite network of patients with impaired amino acid and glucose metabolism was restored. For the research of electro-acupuncture’s effect on RA model rats, Chen and colleagues used ^1^H NMR-based metabolomics.^[79]^ Electro-acupuncture had an effect on the metabolites, which are linked to the metabolic pathway discussed before. Zhu et al^[80]^ and Leng et al^[81]^ (Table 5), for example, collected urine samples from RA model animals and analyzed them using GC-MS in order to better understand the effects of moxibustion on RA. The specificity of moxibustion in the treatment of RA may be explained by changes in the metabolism of amino acids, pyrimidines, and lipids.

**Table 5:** Selected metabolomics studies in bone disease and muscle fatigue

Disease	Model system	Tissue	Platform	Therapy	Acupoint	Metabolic pathway	Findings	Ref
Rheumatoid arthritis	Human	Plasma	GC-MS	Acupuncture	GB20 TE4 LI11 KI10 KI3 SP10 CV4 GB34	Amino acid metabolism; Glucose metabolism	Acupuncture may be able to reestablish the metabolite network associated with amino acid and glucose metabolism.	^[78]^
	Rats	Plasma	^1^H NMR	Electro‑acupuncture	ST36 SJ5	No specific metabolic pathway	Electro-acupuncture has been shown to have a considerable effect on the metabolites of adjuvant arthritic rats, including N-ethylphthalein, glutamine/glutamate, and lactic acid.	^[79]^
	Rats	Urine	GC-MS	Moxibustion	BL23 ST36	Amino acid metabolism; Pyrimidine metabolism; Lipid metabolism	Moxibustion’s specificity in the treatment of rheumatoid arthritis may be reflected in its modulation of amino acid, pyrimidine, and lipid metabolism.	^[80]^
	Rats	Urine	GC-MS	Moxibustion	BL 23 ST 36	TCA cycle; Amino acid metabolism; Purine metabolism; Glucose metabolism	By regulating glycolysis/gluconeogenesis; the TCA cycle; phenylalanine, tryptophan, purine, and butanoate metabolism; and other biological pathways, moxibustion decreased inflammation in rats with arthritis.	^[81]^
Spondylitis	Mice	Serum	UHPLC-Q-TOF/MS	Moxibustion	BL23 ST36 GV4	Lipid metabolism; Purine metabolism; Glucose metabolism; Amino acid metabolism	Moxibustion’s effect on Spondylitis is manifested by changes in metabolites involved in lipid metabolism, purine metabolism, glucose metabolism, amino acid metabolism.	^[82]^
	Mice	Ligament tissue; Urine	UHPLC-Q-TOF/MS	Moxibustion	GV4 BL23 ST36	TCA cycle; Lipid metabolism, Amino Acid metabolism; Purine metabolism.	Moxibustion’s therapeutic benefits on spondylitis may be recognized by 37 endogenous metabolites related to the TCA cycle, lipid metabolism, amino acid metabolism and purine metabolism.	^[83]^
Acute Gouty Arthritis Inflammation	Rats	Urine; Plasma	UPLC-MS	Acupuncture	ST36 SP6	No specific metabolic pathway	Acupuncture may be able to reestablish the metabolite network disrupted by MSU administration.	^[84]^
Delayed-onset muscle soreness	Rats	Muscle	CE-TOFMS	Manual therapy	Left gastrocnemius muscle	Amino Acid metabolism	Manual therapy may be mediated in part by changes in mitochondrial respiration-related metabolites such as branched-chain amino acids, carnitine, and malic acid.	^[85]^
Fatigue Induced by Exhaustive Physical Exercises	Human	Urine	^1^H NMR	Acupuncture	RN8 ST36 CV4 BL40	Energy metabolism; Choline metabolism	Acupuncture treatment alleviated fatigue by re-regulating disrupted energy metabolism, choline metabolism, and attenuating ROS-induced stress at a rapid rate.	^[86]^

GC-MS = gas chromatography-mass spectrometry, NMR = nuclear magnetic resonance, TCA = tricarboxylic acid cycle.

Moxibustion’s impact on spondylitis has also been noted. It was found that moxibustion was effective in alleviating the symptoms of spondylitis in model mice. Metabolites involved in lipid metabolism, purine metabolism, glucose metabolism, amino acid metabolism, and the tricarboxylic acid cycle change as a result of moxibustion treatment for spondylitis.^[82,83]^ In clinical settings, acute gouty arthritis is a common inflammatory disease with several pathogenic mechanisms. Wen et al used UPLC-MS to examine the endogenous metabolites in samples of urine and plasma taken from rats with acute gouty arthritis over the course of several days.^[84]^ For acute gout, acupuncture has been shown to restore the metabolites network that was disturbed.

The discomfort and stiffness felt in muscles after a new or rigorous workout is known as delayed onset muscle soreness. In order to treat musculoskeletal pain, physical therapists employ manual therapy, also known as manipulative therapy, which involves rubbing muscles in which several acupuncture sites are situated. The metabolomics investigation of muscle soreness model animals using capillary electrophoresis coupled to mass spectrometry demonstrates that manual therapy relieves muscular discomfort and modulates amino acid metabolism.^[85]^ In addition, a ^1^H NMR-based metabolomics study was carried out on urine samples from people who had been exhausted as a result of strenuous physical activity.^[86]^ There is evidence to suggest that acupuncture can reduce fatigue after exercise and affect the metabolites involved in energy and choline metabolism.

### 3.5. Other disease

Gynecological illnesses such as primary dysmenorrhea and menopausal syndrome can be effectively treated with herb-partitioned moxibustion and acupoint catgut embedding. Herb-partitioned moxibustion were found to be beneficial in the treatment of abdominal discomfort by Ma et al^[87]^ When it comes to primary dysmenorrhea, acupuncture isn’t as helpful as herb-partitioned moxibustion, which may have something to do with hormone metabolism. In addition, Zhang et al implanted acupoint catgut embedding into a model of menopause in rats.^[88]^ When they stimulated specific acupoints on the animal body, they found that lipid peroxidation was reduced, glucose homeostasis was restored, and the amino acid metabolism was partially reversed. Moreover, during physical exertion that increases abdominal pressure, such as coughing, sneezing, laughing or exercising, stress urinary incontinence may occur. It was found that electro-acupuncture regulated the propanoate, butanoate, and tricarboxylic acid cycles, as well as their relative metabolites in stress urinary incontinence patients. The study used human blood samples from GC-MS based metabolomics approach.^[89]^

TCM has used cupping as an effective treatment since antiquity, and it is now used all around the world. The skin is suctioned using a cup composed of glass, ceramic, bamboo, or plastic. After the cup has been placed on the skin, either a flame is used to remove oxygen or a suction device is added to create negative pressure. Due to the negative pressure created by combustion during cupping therapy, tiny blisters with blood inside (cupping blood) may appear on the skin. The chemicals and metabolic pathways linked with bile acid, glucose and lipid metabolism change significantly between venous and cupping blood samples from humans, according to a ^1^H NMR-based metabolomics study.^[90]^

## 4. Metabolomic characterization of external TCM treatments in various diseases

**Figure 3.**
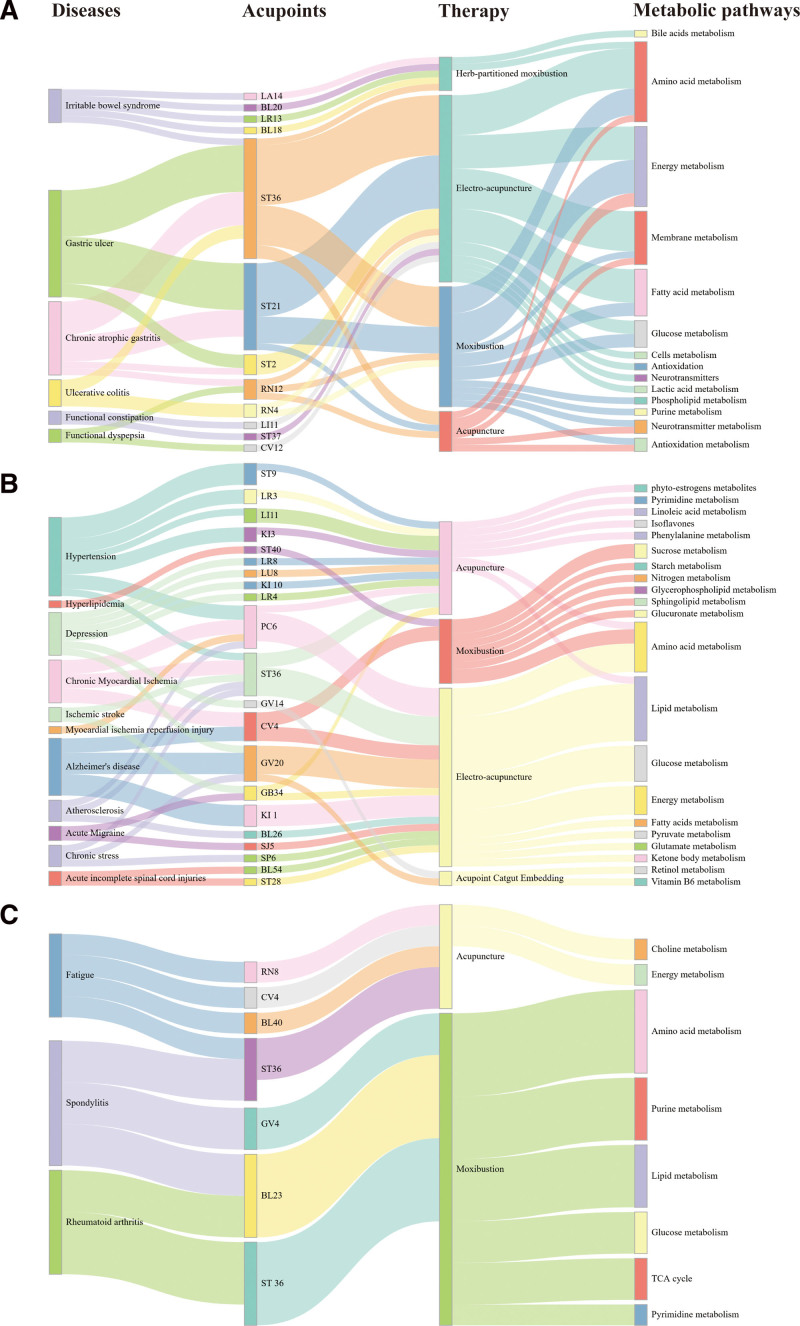
Metabolomic characterization of external TCM treatments in diseases. The Figure A–C summarize the overlapping acupoints, therapy, metabolic pathways in digestive system disease, cardiovascular diseases and neurological disorders, bone disease and muscle fatigue. TCM = traditional Chinese medicine.

In conclusion, the metabolomics studies conducted in the field of external TCM treatments have yielded a substantial number of metabolic pathways that may be implicated in the mechanism of disease amelioration by external TCM treatments. The regulation of metabolomics by the majority of external TCM treatments may be unique to the disease type or sample tissue collected. Nevertheless, a more thorough examination of the metabolomics studies mentioned above, which were applied to external TCM interventions, reveals a minor degree of overlapping metabolic pathway regulation (Fig. 3). Interestingly, different diseases and various modalities of external TCM treatment interventions regulate certain metabolic pathways. These pathways include energy metabolism, lipid metabolism, and amino acid metabolism. Energy metabolism is the comprehensive process by which living cells acquire and utilize the energy required for survival, growth, and reproduction.^[91]^ External treatments in TCM can regulate energy metabolism consistent with its overall regulatory characteristics. Lipid metabolism is the process of oxidizing fatty acids to produce energy or create new lipids from smaller molecules. Lipid metabolism is associated with carbohydrate metabolism, as glucose can be transformed into lipids.^[92]^ Amino acids are vital chemical substances that serve as crucial building blocks for biosynthesis and as a fuel source for living processes.^[93]^ In general, these metabolic pathways are related to the overall process of life activities, and this is in line with the holistic regulation of traditional Chinese medicine.

In summary, ST36 exhibited the maximum frequency in the acupoint selection, and it is often used in combination with ST21 in digestive disorders. As for digestive system disease, electroacupuncture is the most frequently used method, as well as in cardiovascular and neurological disorders. In bone disease and muscle fatigue, moxibustion is the most frequently used method. The choice of different external TCM treatments used in different diseases also demonstrates that although each method works by stimulating meridian and acupoints under the guidance of the basic theories of TCM, they all have their own therapeutic advantages and characteristics. External TCM treatments can ameliorate diseases by regulating multiple metabolic pathways and participating in multiple pathways. It is of great interest to apply metabolomics techniques to explore the mechanisms of external TCM treatments. Moreover, metabolomics technology allows for an in-depth exploration of the differences in mechanisms of action between external TCM treatments.

## 5. Conclusions

Extending TCM external therapy studies to metabolomics was the focus of our investigation in this review. The efficacy of various TCM external treatment techniques has been supported by numerous experimental observations using metabolomics. Metabolomics-based research into TCM’s exterior treatments could be expanded in several areas: An untargeted metabolomics method is used in the majority of current studies. However, TCM’s molecular mechanisms can be supported by more detailed metabolites and metabolic pathways support from targeted metabolomics approach; Acupoint specificity, acupoint compatibility, and clinically effective acupoint prescription should be extensively studied using various targeted metabolomics methods; The metabolic mechanism of non-acupuncture TCM treatments, such as cupping therapy, should be studied more thoroughly. This can better support the choice of the optimal clinical treatment method based on scientific data; and Future research into TCM external therapy should use more multi-omics integrative analysis, such as the integrated study of transcriptomics, proteomics, metabolomics, microbiomics and radiomics. TCM external therapy’s scientific importance can be better understood by integrating several systems biology-related analytical methodologies. In summary, the advancement of the aforementioned research will allow us to gain a deeper understanding of the significance of TCM in the treatment and maintenance of health.

## Author contributions

**Conceptualization:** Xinyue Yang.

**Data curation:** Qingqing Tang.

**Funding acquisition:** Min He, Zhe Wei, Tie Li, Mengmeng Sun.

**Methodology:** Xinyue Yang, Qingqing Tang.

**Project administration:** Tie Li.

**Resources:** Jiazhen Cao.

**Software:** Jiazhen Cao.

**Visualization:** Zhe Wei.

**Writing – original draft:** Xinyue Yang.

**Writing – review & editing:** Xinyue Yang, Min He, Mengmeng Sun.
